# Effect of different doses of nitrogen fertilization on bioactive compounds and antioxidant activity of brown rice

**DOI:** 10.3389/fnut.2023.1071874

**Published:** 2023-02-03

**Authors:** Yichao Ma, Shuang Zhang, Daguang Feng, Nuoqi Duan, Liyan Rong, Zhaoxia Wu, Yixiao Shen

**Affiliations:** ^1^College of Food Science, Shenyang Agricultural University, Shenyang, China; ^2^College of Science, Shenyang Agricultural University, Shenyang, China

**Keywords:** brown rice, nitrogen fertilization, phenolic acid, antioxidant, predictive models

## Abstract

Brown rice as a whole grain food is associated with various chronic diseases’ reduced risks. In this study, the effects of different doses of nitrogen fertilization (0, 160, 210, 260, 315, and 420 kg N/ 100 m^2^) on bioactive compounds and antioxidant activity of brown rice (yanfeng47) were investigated. At nitrogen level of 210–260 kg N/100 m^2^, the content of TFC (302.65 mg/100 g), β-sitosterol (1762.92 mg/100 g), stigmasterol (1358.735 mg/100 g), DPPH (74.57%), and OH free radical scavenging (74.19%) was the highest. The major phenolic acid was p-hydroxybenzoic acid. There were significant positive linear relationships between TFC (0.872, 0.843), β-sitosterol (0.896, 0.657), stigmasterol (0.543, 0.771), p-hydroxybenzoic acid (0.871, 0.875), and DPPH, OH antioxidant activity. These indicated that TFC and phytosterols were the most important components in brown rice that had strong antioxidant activity. Composite score of principal components indicated 210 Kg N/100 m^2^ exhibited a more ideal dose of nitrogen for nutritional composition and antioxidant activity of brown rice.

## 1. Introduction

Nowadays, scientists, governments, health groups, and consumers all over the world are becoming interested in the whole cereal grain, because it has high bioactive chemicals and health advantages (1). Brown rice, commonly known as whole grain rice, is a reliable source of food for people’s daily consumption. Brown rice contains many bioactive phytochemicals, such as carotenoids, anthocyanins, proanthocyanidins, tocopherols, sitosterol, GABA (gamma-aminobutyric acid), and other phenolic compounds (2). Consumption of whole grain products has been linked to a lower risk of numerous chronic diseases in recent epidemiological research (3). Brown rice has been demonstrated in numerous studies to have antioxidant, anti-inflammatory, anticancer, neuroprotective, and cholesterol-lowering properties (4–6). It also reduces the danger of metabolic disorders and cardiovascular diseases (7). However, investigation on the health benefits of phytochemicals about brown rice, such as phenolic and flavonoid, is limited. Therefore, it is necessary to systematically study the variation of bioactive phytochemicals in brown rice.

The bioactive compound content of brown rice varied according to rice variety, cultivation environment, harvesting time, and fertilization rates. In the same growing region, studies show that fertilizer is the key to the rice production (8, 9). Meanwhile, nitrogen is the most important nutrient element for plant growth and production (10). Proper application of nitrogen fertilizer is an effective practice to improve plant growth, crop yield and quality (11). Nitrogen fertilizers have a significant impact on rice eating and cooking quality (12). Liang et al. (12) showed that increasing nitrogen fertilizer could improve the rice processing quality. Furthermore, nitrogen fertilization has varied impacts on the concentration of bioactive composition in various crop species. *Lycium Barbarum* improved betaine production with increasing nitrogen fertilizer treatment (13). In the yaupon, nitrogen application can increase the contents of caffeine and phenolic but had no change on its flavonoid (14). In wheat grains, bioavailability of phenolic compounds rose with increasing nitrogen supply (15). However, the association between nitrogen fertilizer amount and rice bioactive chemicals remains uncertain. Currently, most research on the impacts of rice fertilization has concentrated on rice yield and quality, rather than the bioactive phytochemicals.

Brown rice is also available as complementary edible rice. It is affected by many factors in the actual production process, but fertilizer application is the most important element that can be managed by humans. Since we selected the main species for the study in the Panjin area of Liaoning, which is mainly saline, nitrogen fertilizer has a greater impact on rice than phosphorus and potassium fertilizers. As a result, we evaluated the changes in phytochemicals and antioxidant activity of brown rice with various amount of nitrogen fertilization in the current study. The purposes of this study were to help improve the nutritional value of edible brown rice by suggesting the application of an appropriate amount of fertilizer in agricultural production. Furthermore, we attempted to find a linear relation between nitrogen fertilization and antioxidant activity.

## 2. Materials and methods

### 2.1. Materials

The test area of the present study was conducted at Liaohe Delta, Panjin, Liaoning Province, China (122° 14′ 17″ N, 41° 9′ 31″ E). The soil parameters were tested in advance with method by Liu et al. (16). The physical and chemical properties of the experimental soil are shown in Table 1. The sample used was Yanfeng 47, which is one of the main local varieties. The local planting conditions in growing season in 2020 are: average temperature −4.9°C, average annual precipitation of 30.8 mm, and average sunshine of 561.5 h. The seedlings were transplanted on 25 May and were harvested on 8 October in 2020. Based on the previous study (Supplementary Table 1), the conditions of nitrogen application (0, 160, 210, 260, 315, and 420 kg N/ 100 m^2^) were set. The dispersion was a control group without nitrogen treatment (0 kg N/100 m^2^) and five treatment groups with nitrogen treatment (160, 210, 260, 315, and 420 kg N/100 m^2^). The experiment was conducted in three biological replicates.

**TABLE 1:** Physical and chemical properties of experimental soil.

Soil pH	Organic matter (mg/kg)	Total nitrogen (g/kg)	Available nutrient (mg/kg)			Bulk density (g/cm^3^)
			**N (nitrogen)**	**P (phosphorus)**	**K (potassium)**	
8.2	22.57	1.42	105.24	21.61	164.22	1.39

### 2.2. Rice sample and sample preparation

All harvested grains were air-dried for a month to reduce moisture content to approximately14%. The brown rice was polished using a rice milling machine (Satake Co., Hiroshima, Japan) to obtain approximately 10% (w/w) rice husk and approximately 90% (w/w) rice. The brown rice samples were ground to powder completely through an 80-mesh sieve, store at room temperature.

### 2.3. Determination of total phenolic (TPC)

Total phenolic content (TPC) was determined by the Folin-Ciocalteu colorimetric method described previously (17). Briefly, 100 μL of sample extractor was treated with 100 μL of Folin-Ciocalteu reagent for 6 min. Afterward, the mixture was alkalinized with 1 mL of 7% Na_2_CO_3_. After being kept in dark for 90 min, the mixture was measured at 760 nm. This step was repeated three times. TPC of each sample was presented as mg GAE per 100 g dry weight (DW).

### 2.4. Determination of total flavonoid content (TFC)

Currently, the determination of TFC was depended on the aluminum chloride colorimetric method described by Min et al. (18). The absorbance of each samples was at 510 nm. Standard rutin curve was used to calculated the content of TFC. Total flavonoid content was recorded as mg of RE/100 g dry weight (DW).

### 2.5. Determination of phenolic acid content

The determination of phenolic acid content was performed on HPLC as described previously (19). The mobile phase for separation of phenolic acid was made up with 0.05% trifluoroacetic acid in water (A) and methanol (B). Sample extract was injected with a speed of 1 mL/min. The phenolics contents were represented as mg/100 g DW.

### 2.6. Determination of phytosterol

Take 3.0 g sample was placed in a conical flask and added with anaqueous ethanol solution for Ultrasonic extraction for 40 min. 50% NaOH solution was added at a volume ratio of 1: 1, saponified in a water bath at 80°C for 60 min. After cooling, 10 mL of water was added, and then transferred to the separator funnel. 15 mL dichloromethane was extracted three times, combined with the extraction solution, spin dried, and 2 mL methanol was used. For resolution, use 0 22 μm microporous membrane filtration for HPLC injection.

The UV scanning of two phytosterol standard solutions at 190 ∼400 nm showed that stigmasterol and β-sitosterol had the strongest absorption at 205 nm, so the detection wavelength was selected at 205 nm. Waters ACQUITY UPLC BEH C8 (2.1 mm × 100 mm, 1.7 μm) column was used at a flow rate of 0.4 mL/min. The column temperature was 35°C, and the sample size was 10 μL. Mobile phase: phase A was water, phase B was acetonitrile, gradient elution, 0–9.5 min 80–90% B phase.

### 2.7. Determination of antioxidant activity

#### 2.7.1. Determination of DPPH radical scavenging activity

The method described by Qiu et al. (20) was used with slight modifications to assess DPPH of each extract. The mixtures are shaken vigorously and taking the sample represented by Ai is incubated 30 min in the dark. Mixture was measured at 517 nm. The calculation formula as follows:


$$\text{DPPHradical}\mathrm{scavenging}\mathrm{effect}\left(\%\right)=1-\frac{A_{s\,a\,m\,p\,l\,e}-A_{b\,a\,c\,k\,g\,r\,o\,u\,n\,d}}{A_{c\,o\,n\,t\,r\,o\,l}}\times 100\%$$

where A_sample_, A_control_, and A_background_ refer to the absorbance of the sample solution (sample and DPPH), control solution (without sample), and background solution (without sample), respectively.

#### 2.7.2. Determination of hydroxyl radical scavenging activity

The method described by Min et al. (18) and Qiu et al. (20) was used with slight modifications to assess the hydroxyl radical scavenging activity of each extract. A 1.0 mL anhydrous ethanol solution with a concentration of 1.865 mmol/L phenanthroline monohydrate., and the samples with phosphate buffer pH 7.4. The mixtures are shaken vigorously. 1.0 mL 1.865 mmol/L FeSO_4_ solution was mixed again. Then 1.0 mL 0.03% H_2_O_2_ was added into 37°C water, 60 min. The absorbance measured at 536 nm. Hydroxyl radicals were obtained by interpolation from linear regression analysis.


$$\mathrm{scavenging}\mathrm{effect}\left(\%\right)=\frac{A_{S}-A_{n}}{A_{b}-A_{n}}\times 100\%$$

where A_n_, A_s_, and A_b_ refer to the absorbance of the control solution (water replace H_2_O_2_), sample solution, and background solution (without sample), respectively.

### 2.8. Statistical analyses

All measurements in this study were presented as means ± standard deviations. Each antioxidant activity assay was carried out three times from the same extracts in order to determine their reproducibility. Statistical differences and principal component analysis were analyzed with SPSS software (21).

## 3. Results

### 3.1. The effect of nitrogen fertilization on total phenolic content (TPC)

**FIGURE 1:**
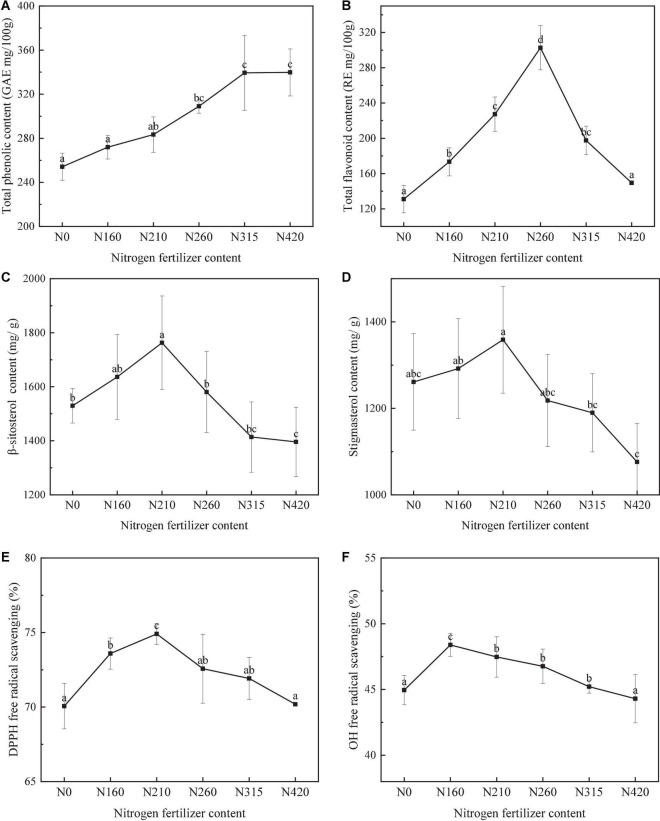
Effect of different amounts of nitrogen fertilization on the total phenolic **(A)**, flavonoids **(B)**, β-sitosterol **(C)**, stigmasterol **(D)**, DPPH **(E)**, and OH **(F)** content of brown rice (lowercase represent differences between groups, *P <* 0.05).

The total phenol content (TPC) values of brown rice in different nitrogen fertilizer application (0, 160, 210, 260, 315, and 420 kg/100 m^2^) are shown in Figure 1. Total phenolic content ranged from 254.12 to 339.77 mg/100 g of brown rice. TPC was significantly affected by different nitrogen fertilizer applications (*P* < 0.05). At level of 260 kg/100 m^2^, the highest content was 348.49 mg/100 g. The contents of free and bound phenols reached the highest at 260 kg/100 m^2^, which were 122.34 and 226.15 mg/100 g, respectively. The contribution of bound phenols to total phenols was higher than 60% at different fertilizer application levels, and it was as high as 68.07% at 160 kg/100 m^2^. It could be because nitrogen application increases the TPC of brown rice. However, the total phenolic content decreased slightly in 420 kg/100 m^2^.

### 3.2. The effect of nitrogen fertilization on total flavonoid content (TFC)

**FIGURE 2:**
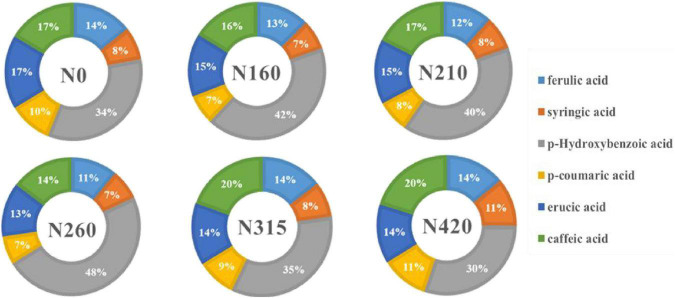
Effect of different amounts of nitrogen fertilization on phenolic composition of brown rice.

The total flavonoid content (TFC) in different nitrogen fertilizer application is shown in Figure 1. The TFC values of brown rice varied significantly (*P* < 0.05), ranging from 130.96 to 302.65 mg/100 g (Figure 2). Then, the highest content of 302.65 mg/100 g was found at 260 kg/100 m^2^. However, the trend of TFC in different nitrogen fertilizer application was not consistent with the trend of total phenolic content. When nitrogen application rate was over 260 kg/100 m^2^, TFC was significantly decreased. The contents of bound flavonoid reached the highest at 260 kg/100 m^2^, which were 228.28 mg/100 g. The contribution of bound phenols to total phenols was higher than 55% at different fertilizer application levels, and it was as high as 75.07% at 260 kg/100 m^2^. The effect of different nitrogen application rate on bound flavonoids was greater than that of free.

### 3.3. The effect of nitrogen fertilization on phenolic composition

Six phenolic compounds, including ferulic acid, syringic acid, p-hydroxybenzoic acid, p-coumaric acid, erucic acid and caffeic acid were determined. The individual phenolic content in rice, as well as the percentage contribution to the total content, are shown in Table 2 and Figure 2. The effect of nitrogen fertilization on ferulic acid was significant (*P <* 0.05). Ferulic acid content declined dramatically as nitrogen fertilization increased. However, the trends of syringic acid and erucic acid were similar at different nitrogen fertilization. This could be owing to chemical structure, that one hydrogen bond on the benzene ring has been replaced by dimethoxy in both of them. It is noteworthy that the level of syringic acid and erucic acid dropped at 160 kg/100 m^2^. The highest concentrations were 8.10 and 15.46 mg/100 g, respectively, at 210 kg/100 m^2^. However, hydroxybenzoic acid, p-coumaric acid, and caffeic acid, as derivatives of cinnamic acid, increased in content at less than 210 kg/100 m^2^, while the content decreased significantly, above 260 kg/100 m^2^. All three phenolic acids was found the same trend in different doses of nitrogen fertilization. Therefore, we hypothesize that the influence of different nitrogen fertilization levels on the content of individual phenolic acids is connected to their structures. From the Figure 2, it shows that p-hydroxybenzoic acid is the main phenolic acid in brown rice. Under high nitrogen application, the proportions of ferulic, erucic acid and caffeic acid in total phenolic acids remained stable, whereas syringic acid and p-coumaric acid proportions increased and p-hydroxybenzoic acid declined. This suggests that syringic acid, p-coumaric acid, and p-hydroxybenzoic acid were more sensitive to high nitrogen levels. In contrast, the proportions of syringic acid and p-coumaric acids were almost unchanged under low nitrogen application. When the nitrogen level was lower than 210 kg/100 m^2^, the proportions of ferulic, erucic, and caffeic acid decreased significantly, while the proportion of p-hydroxybenzoic acid increased significantly. This suggests that different phenolic acid species react to nitrogen levels in distinct ways. At 210 kg/100 m^2^, the cumulative level of the six phenolic acids was the greatest.

**TABLE 2:** Phenolic composition of brown rice.

Nitrogen	Ferulic acid	Syringic acid	Hydroxybenzoic acid	P-coumaric acid	Erucic acid	Caffeic acid
N0	12.25 ± 1.21^a^	6.59 ± 0.51^b^	29.13 ± 3.29^a^	5.73 ± 0.38^a^	14.76 ± 1.50^ab^	14.01 ± 1.39^b^
N160	11.85 ± 1.09^b^	6.29 ± 0.47^b^	37.89 ± 1.91^b^	6.36 ± 0.40^c^	13.25 ± 1.32^b^	14.41 ± 1.44^b^
N210	11.15 ± 1.17^ab^	8.10 ± 0.69^a^	40.90 ± 2.28^b^	7.83 ± 0.32^b^	15.46 ± 1.59^a^	18.01 ± 1.88^a^
N260	7.65 ± 0.65^bc^	4.99 ± 0.32^c^	33.89 ± 1.42^b^	4.71 ± 0.24^d^	8.95 ± 0.79^c^	10.30 ± 0.91^c^
N315	7.55 ± 0.63^bc^	4.79 ± 0.23^c^	17.86 ± 0.56^b^	4.68 ± 0.30^d^	7.44 ± 0.61^cd^	10.10 ± 0.94^c^
N420	5.94 ± 0.44^c^	4.29 ± 0.29^c^	12.85 ± 1.16^b^	4.38 ± 0.02^d^	5.84 ± 0.41^d^	8.60 ± 0.73^c^

Lowercase letters represent differences between groups (*P* < 0.05).

### 3.4. The effect of nitrogen fertilization on phytosterols

#### 3.4.1. The effect of nitrogen fertilization on β-sitosterol content

The variation of β-sitosterol content of brown rice is shown in Figure 1. The effect of nitrogen fertilization on brown rice was significant (*P <* 0.05). With increased nitrogen fertilization, the concentration of -sitosterol increased, then decreased, with the highest content of 1762.92 mg/g at 210 kg/100 m^2^. This indicated that nitrogen fertilization can promote the increase of active matter in brown rice to some extent. It’s possible that it’s because nitrogen fertilization affects its bioactive enzymes, and high nitrogen inhibits enzyme activity during rice growth.

#### 3.4.2. The effect of nitrogen fertilization on stigmasterol

The Figure 1 showed the stigmasterol content in different nitrogen fertilizer application of brown rice. Stigmasterol content was significantly affected by different nitrogen fertilization (*P <* 0.05). The highest stigmasterol content was 1359.21 mg/g at 210 kg/100 m^2^. Then, when nitrogen application increased, the stigmasterol content decreased with a maximum of 25.81%. This suggested that fertilizing with the right quantity of nitrogen can assist to boost stigmasterol levels.

### 3.5. The effect of nitrogen fertilization on antioxidant

In order to evaluate the antioxidant properties of different rice, two assays including DPPH and OH free radical scavenging were used. The assay of radical scavenging activity against DPPH radicals is a frequently used approach to measure antioxidant activity, although it focuses on the assay of hydrogen-donating antioxidants against nitrogen radicals. Then, the antioxidant theories used in the assay are OH and DPPH free radical scavenging.

In different nitrogen fertilization, DPPH free radical scavenging of brown rice is displayed in Figure 1. The DPPH free radical scavenging activities ranged from 70.06 to 74.57%, varied significantly (*P <* 0.05). With increasing amount of nitrogen, the trend of DPPH free radical scavenging activities was consistent with of the levels of flavonoids and β-sitosterol.

In different nitrogen fertilization, hydroxyl free radical scavenging of brown rice is displayed in Figure 1. The hydroxyl free radical scavenging activities varied significantly from 42.03 to 74.19% (*P <* 0.05). With the nitrogen fertilization increasing, the OH free radical scavenging activities was increased and then decreased quickly. The highest content of hydroxyl free radical scavenging activity of 74.19%. Was found at 210 kg/100 m^2^.

### 3.6. Correlation analysis

To assess the correlation between total phenolic, flavonoids, phytosterols, and phenolic acids and the antioxidant activity of rice with different nitrogen fertilization, the relationship between these contents and the antioxidant activity was analyzed (Table 3). The DPPH scavenging activities had significantly correlation with flavonoids (0.872), β-sitosterol (0.896) and p-hydroxybenzoic acid (0.871). The OH scavenging activities had significantly correlation with flavonoids (0.843), stigmasterol (0.771) and p-hydroxybenzoic acid (0.875). Flavonoids and p-hydroxybenzoic acid were the major contributors to the antioxidant activity of brown rice. β-sitosterol and stigmasterol exhibited different antioxidant capacities, probably due to the different mechanisms of action of DPPH and OH in determining the antioxidant capacity.

**TABLE 3:** Correlation analysis between bioactive compounds and the antioxidant activity.

	Phenolic	Flavonoids	β-sitosterol	Stigmasterol	Ferulic acid	Syringic acid	Hydroxybenzoic acid	p-coumaric acid	Erucic acid	Caffeic acid
DPPH	0.486	0.872*	0.896**	0.543	0.557	0.787	0.871*	0.547	0.257	0.600
OH	-0.429	0.843*	0.657	0.771*	0.468	0.429	0.875*	0.143	0.543	0.714

*Represent differences (*P* < 0.05), ** (*P* < 0.01).

### 3.7. Principal component analysis (PCA)

The PCA method was used to transform multiple and interrelated indicators into new, fewer and independent composite indicators. In this study, PCA was conducted on bioactive ingredients and antioxidant activity were shown in Table 4.

**TABLE 4:** Principal component analysis.

Component	Starting eigenvalue			Retrieve square and load		
	**Total**	**Variable %**	**Cumulative %**	**Total**	**Variable %**	**Cumulative %**
1	5.980	59.804	59.804	5.980	59.804	59.804
2	1.650	16.497	76.301	1.650	16.497	76.301
3	1.092	10.916	87.217	1.092	10.916	87.217
4	0.792	7.916	95.133			
5	0.327	3.267	98.400			
6	0.130	1.296	99.696			
7	0.023	0.232	99.928			
8	0.006	0.056	99.984			
9	0.001	0.013	99.997			
10	0.000	0.003	100.000			
11	5.980	59.804	59.804			
12	1.650	16.497	76.301			

The selection of the principal component should be done in conjunction with the dimension reduction principle. The accumulated random contribution rate was no less than 80% in determining the number of primary components (22). The analysis showed the cumulative contribution rate of the first three reached 87.217%, the first principal component explained 59.804%, the second explained 16.497%, and three explained 10.916%. Therefore, the cumulative contribution rate could be used to explain the effect of different amounts of nitrogen fertilization on bioactive components and antioxidant activity of rice.

In this case, phytosterols and phenolic acid were closely related antioxidant activity. From the previous correlation analysis, among phenolic acids, the p-hydroxybenzoic acid is more correlated with the DPPH radical (0.871). Therefore, it is mainly responsible for preventing the oxidation mechanism promoted by oxygen radicals. Profiles of flavonoids and phytosterol found in rice samples better reinforce their antioxidant activity, rather than their total content of phenolic. Accordingly, it is for the most part answerable for forestalling the oxidation component advanced by oxygen revolutionaries.

#### 3.7.1. Scores for different components

The eigenvectors of the first three principal components were calculated based on the principal component loading matrix and the eigenvalues. The ratio of the eigenvalues corresponding to each principal component to the sum of the total eigenvalues of the extracted principal components was used as the weight to calculate the integrated principal component value (Table 5). It indicated that the nitrogen fertilization amount of 210 kg/100 m^2^ exhibited a more ideal balance between nutritional composition and antioxidant activity. Moderate fertilization can maintain the phytochemical active components and antioxidant activity of brown rice effectively.

**TABLE 5:** Scores of brown rice.

Nitrogen	Brown rice	
**(kg/100 m^2^)**	**Score**	**Comprehensive ranking**
0	−0.84316	5
160	−0.77368	3
210	−0.67370	1
260	−0.72564	2
315	−0.83468	4
420	−0.87012	6

### 3.8. Predictive model

The mathematical model was derived by exploring the relationship between antioxidant activity and the amount of nitrogen fertilization based on the three principal components derived from the principal component analysis. The model equation is shown below:


DPPH%=70.147+0.011N+2.292X1+1.049X2

$$\;-2.383\text{X3}\left(R^{{}^{2}}=0.836\right).$$

$$\mathrm{OH}\%=75.077-0.057N-3.262\mathrm{X1}-0.4\mathrm{X2}-3.289\text{X3}\left(R^{{}^{2}}=0.884\right).$$

X1,X2,X3– The three factors derived for the principal components.

N– the amount of nitrogen fertilization.

Since the antioxidant mechanism of action was different, the amount of nitrogen fertilization has different effects on the antioxidant activities. Using the formula, the amount of nitrogen fertilization could be adjusted to increase the bioactive content.

## 4. Discussion

In this current study, in anticipation of improving the nutritional value and antioxidant activity of brown rice, controlling the nitrogen fertilization was an effective application. The results demonstrated a linear association between the amount of nitrogen fertilizer applied and the level of bioactive compounds or antioxidant capacity of brown rice. Brown rice demonstrated high TPC, TFC, β-sitosterol, and stigmasterol contents, as well as the activity of scavenging DPPH and OH values which varied with the fertilization of different nitrogen levels. DPPH scavenging activity was also greater than some weedy species (23, 24). The contents of TPC and TFC in different plants are varied. Since TPC and TFC have antioxidant activities, they will also affect the antioxidative capacity of plants. The phytochemicals with increased nitrogen fertilizer was linked to bioactive substance synthesis pathways (25). Mir et al. (26) demonstrated that a moderate nitrogen fertilizer rate can boost rice output during actual production. As a result, this study discovered that increasing the amount of nitrogen fertilizer applied to brown rice could improve its bioactive content and antioxidant capability while maintaining rice yield. This discovery has significant implications for increasing the nutritional content of brown rice by regulating nitrogen fertilizer.

Brown rice’s phenolic components have been demonstrated in numerous studies to have antioxidant activity and possible health benefits. Phenolic acids are mainly metabolized through phenylalanine and tyrosine during rice growth, and high nitrogen conditions may inhibit such amino acids, which in turn affect the phenolic acid content in brown rice (27). Palafox-Carlos et al. (28) reported that the interactions among phenolic acids in a mixture can affect total DPPH antioxidant activity. Rasera et al. (29) reported that the interactions among phenolic acids can affect antioxidant capacity. However, the results of this experiment showed that flavonoid are the main antioxidant substances in brown rice. The main contributions to brown rice’s antioxidant activity, were flavonoid and phytosterols according to correlation research. This result was consistent with previous work of Xya et al. (30)’s and Yu et al. (31)’s study that showing a high correlation between phenolic acid content, total flavonoid content and antioxidant activities determined by DPPH (0.781, 0.856).

In recent years, brown rice (BR) has gained growing attention due to possess various nutritionally dense bioactive phytochemicals. It has higher concentration of beneficial chemicals than white rice. As scientific proof of wholegrains’ health benefits grows, dietary guidelines around the world are recommending their inclusion in the diet. According to the Food and Agriculture Organization of the United Nations and the World Health Organization Nutrition Conference, the United States recommended eating whole grain foods and eating more than three cups of cheese per day. Italy and Poland suggested substituting whole-grain bakery products for white-flour bakery products, and Greece suggested substituting whole-grain bakery products for white-flour bakery products. The Netherlands asked for at least 90 grams of brown or whole wheat bread per day, and Denmark asked for at least 75 grams of whole grains per day. Brown rice consumption is becoming more popular in Asian populations where take rice as the main food (32). The recommended daily consumption of cereals is 200–300 g, and whole grain meals is 50–100 g, according to Dietary Guidelines for Chinese Residents (33). In this study, total phenolic and flavonoid content in whole grain foods was 254.12–339.77 mg/100 g and 130.96–302.65 mg/100 g, respectively, therefore each person would consume around 60–300 mg of phenols and flavonoids per day. These studies investigating the health-beneficial substance in brown rice have prompted to advance whole grain utilization instead of white grains.

In addition, Imam et al. (34) showed that brown rice as dietary components can guard against total antioxidant status deteriorated in type 2 diabetic rats. As a result of this study, 150–200 g rats were chosen for a 2-week treatment with a daily consumption of 15–20 g. Although human physiology differs from that of rodents, it is possible that consuming whole grains over a lengthy period of time according to Dietary Guidelines will help people prevent type 2 diabetes. Therefore, if brown rice is consumed whenever possible, it can improve the antioxidant capacity of the body and reduce the risk of chronic diseases. However, the mechanism of how whole grain cereal affects health is still being studied at present. We will consider an in-depth study of the health mechanism in the next study.

## 5. Conclusion

The presented research described a systematic evaluation of the effects of the nitrogen fertilization on bioactive constituents (TPC, TFC, phytosterols, phenolic acids) as well as antioxidant activity (DPPH, OH) of brown rice. Significant differences of nitrogen fertilization were observed in TFC, phytosterols and antioxidant capacity of brown rice. Correlation analyses confirmed that TFC, β-sitosterol and stigmasterol significantly contributed to the antioxidant activity. It is noteworthy that p-hydroxybenzoic acid is the main phenolic acid in brown rice. Furthermore, the concentrations of six phenolic acid compounds varied dramatically depending on the amount of nitrogen fertilization applied. The nitrogen fertilizer quantity of 210 kg/100 m^2^ has a more optimum nutritional composition, according to the composite ratings of primary components. Based on these findings, it is recommend that nitrogen fertilization be carefully regulated throughout rice processing to optimize both bioactive components and antioxidant activity. This research provides important evidence that fertilization can improve biological activity, and presents a model of the relationship between antioxidant and nitrogen application.

## Data availability statement

The original contributions presented in this study are included in the article/Supplementary material, further inquiries can be directed to the corresponding authors.

## Author contributions

YM: conceptualization, methodology, and writing—original draft. SZ: investigation and supervision. YS: writing—review and editing. DF: data curation and software. ND and LR: methodology. ZW: visualization and validation. All authors contributed to the article and approved the submitted version.
